# Texture, Nutrition, and Flavor of Different Freshwater Fish Muscles: Comparative Study and Molecular Docking

**DOI:** 10.3390/foods14132258

**Published:** 2025-06-26

**Authors:** Banghua Xia, Jiaming Zhang, Chenhui Li, Song Wu, Li Huang, Dongli Qin, Qirui Hao, Lei Gao

**Affiliations:** 1 Heilongjiang River Fisheries Research Institute, Chinese Academy of Fishery Sciences, Harbin 150070, China; xbh26@sdu.edu.cn (B.X.); lichenhui@hrfri.ac.cn (C.L.); wusong@hrfri.ac.cn (S.W.); huangli@hrfri.ac.cn (L.H.); qindongli@hrfri.ac.cn (D.Q.); 2 Marine College, Shandong University, Weihai 264209, China; blue_jmzhang@163.com; 3 Supervision, Inspection and Testing Center for Fishery Environment and Aquatic Products (Harbin), Ministry of Agriculture and Rural Affairs, Harbin 150070, China; 4 Laboratory of Quality and Safety Risk Assessment for Aquatic Products (Harbin), Ministry of Agriculture and Rural Affairs, Harbin 150070, China; 5 College of Animal Science and Technology, Northeast Agricultural University, Harbin 150030, China

**Keywords:** HS-SPME-GC-MS, electronic nose, TPA, nutritional composition, molecular docking

## Abstract

*Cyprinus carpio*, *Parabramis pekinensis*, *Aristichthys nobilis*, and *Lateolabrax maculatus* were systematically evaluated as crucial components of Chinese aquaculture with substantial market demand. Texture profile analysis (TPA) showed *C. carpio* had maximal hardness, while *L. maculatus* displayed optimal elasticity. Nutrient composition analysis revealed that the highest crude protein content was identified in *L. maculatus*, while a higher crude lipid level was recorded in *C. carpio*. Fatty acid profiling established *L. maculatus* as a superior source of monounsaturated fatty acids (MUFAs), whereas *P. pekinensis* was distinguished by its polyunsaturated fatty acid (PUFA) content. Volatile compounds were comprehensively analyzed using an electronic nose (e-nose) coupled with HS-SPME-GC-MS, resulting in the identification of 59 flavor compounds. Molecular docking demonstrated that hydrogen bonding and π–π stacking were identified as critical mechanisms governing flavor perception. These findings offer valuable information that can support improvements in aquaculture management practices and help inform consumer choices regarding fish quality.

## 1. Introduction

The Heilongjiang River Basin in northeastern China enjoys ample rainfall from the temperate monsoon climate and water retention from high-latitude permafrost, providing excellent conditions for freshwater fish growth and reproduction [1]. The natural waters of the Heilongjiang River Basin are home to 105 species of fish, accounting for 13% of China’s freshwater fish species [2]. Among them, *Cyprinus carpio*, *Parabramis pekinensis*, *Aristichthys nobilis*, and *Lateolabrax maculatus* are the main bulk freshwater fish species of the Heilongjiang River Basin and are highly favored by consumers.

Fish muscle quality is closely related to feeding habits, as well as species, regional climate, and living conditions [3]. *C. carpio* feeds on mollusks such as mussels and clams, as well as aquatic insect larvae, small fish, and shrimp [4]. *P. pekinensis* mainly feeds on algae, aquatic insect larvae, and small amounts of other aquatic plant fragments [5]. *A. nobilis* is a classic zooplankton feeder. In captivity, they also consume artificial feeds like soybean meal, rice bran, and distiller’s grains [6]. Juvenile *L. maculatus* mainly feed on aquatic insects, small shrimp, and small fish, while adults primarily prey on other fish [7]. These different living habits and feeding patterns result in variations in fish meat quality among the four freshwater fish species. However, there is still a lack of systematic research on the muscle quality and flavor profiles of these four freshwater fish species.

The muscle quality of farmed fish can be evaluated from three perspectives: texture, nutritional composition, and flavor. Texture is defined as the mechanical or rheological properties influenced by tissue structure and composition [8]. Currently, texture profile analysis (TPA) is one of the commonly used methods for texture analysis [9]. It employs instruments to simulate chewing behavior and evaluate muscle samples, effectively reflecting muscle quality and sensory acceptability by eliminating subjective bias. In terms of muscle nutritional composition, current research mainly emphasizes basic nutrients, amino acid profiles, and fatty acid profiles.

The flavor of freshwater fish primarily originates from volatile compounds, umami amino acids, and lipid oxidation products, and is influenced by multiple factors, including species, feed composition, water environment, farming methods, and processing treatments [10,11,12]. For instance, a study demonstrated that histidine improved the amino acid composition and flavor of grass carp (*Ctenopharyngodon idella*) muscle [13]. Additionally, short-term purification significantly improved the fishy odor in crucian carp (*Carassius auratus*) [14]. With the development of techniques such as GC-MS, electronic nose, and metabolomics, the detection and analysis of flavor compounds have become more precise, providing technological support for improving fish meat flavor quality through strategies like nutritional regulation, breeding optimization, and ecological farming [15,16,17,18].

Human olfactory receptors (ORs) are a class of G protein-coupled receptors (GPCRs) related to olfaction, capable of recognizing various chemical molecules and transmitting olfactory signals to the brain [19]. Molecular docking serves as a pivotal tool for analyzing interactions between olfactory receptors (ORs) and odorant molecules, providing critical insights into recognition mechanisms between food components and sensory receptors [20]. However, research on the binding patterns of volatile flavor compounds in freshwater fish muscle to ORs remains limited.

In recent years, there have been some studies exploring the muscle quality of freshwater fish. However, the lack of complex and systematic extraction and separation equipment and methods, as well as differences in fish sampling specifications, timing, and geographic locations, have resulted in the omission of many important muscle quality parameters. Therefore, this study systematically analyzed and compared the quality and flavor of *C. carpio*, *P. pekinensis*, *A. nobilis*, and *L. maculatus*. The results clarify interspecific differences in freshwater fish muscle quality and support the selection and breeding of high-quality varieties for fisheries and consumers.

## 2. Materials and Methods

### 2.1. Experimental Sample

Ten individuals each of *C. carpio*, *P. pekinensis*, *A. nobilis*, and *L. maculatus*, all of uniform size (1000 ± 10 g), healthy, unscarred, and active, were randomly selected in June 2024. All fish were obtained from the Heilongjiang Fishery Research Institute of the Chinese Academy of Fishery Sciences (Harbin, China) and were cultured under standardized aquaculture conditions. Prior to sampling, all fish were reared in the same recirculating aquaculture system with a controlled water temperature of 22 ± 1 °C, dissolved oxygen above 6.0 mg/L, and pH maintained at 7.2–7.6. The fish were fed a commercial diet twice daily and fasted for 24 h before sampling to minimize variability due to recent feeding.

To ensure consistency in physiological state, all individuals were of similar age (approximately 1 year) and were farmed under identical conditions. No wild specimens were used in this study to control for environmental and dietary variability. Fish were transported in oxygenated bags to the College of Animal Science and Technology at Northeast Agricultural University (Harbin, China) and allowed to acclimate for 12 h before processing.

Fish were fully anesthetized using 3-aminobenzoic acid ethyl ester methanesulfonate (purity > 99%, CAS: 886-86-2, AbMole Bioscience Co., Ltd., Shanghai, China) at a concentration of 250 mg/L, followed by euthanasia in accordance with ethical guidelines. Muscle samples (15 ± 0.05 g) were aseptically collected from both sides of the dorsal region using sterile scalpels for texture analysis. The remaining dorsal muscle was flash-frozen in liquid nitrogen and stored at –80 °C for subsequent nutritional and flavor analyses. All procedures were approved by the local ethics committee and complied with the European Directive 2010/63/EU for animal experiments and were performed following relevant FAO (Food and Agriculture Organization) and AOAC (Association of Official Analytical Chemists) guidelines for biological sampling and sample preparation. Furthermore, heavy metals, including mercury (Hg), lead (Pb), and cadmium (Cd), in dorsal muscle samples were analyzed using inductively coupled plasma mass spectrometry (ICP-MS, Agilent 7900, Agilent Technoloaies, Santa Clara, CA, USA), and none of these elements were detected in any of the samples.

### 2.2. Basic Physicochemical Analysis

#### 2.2.1. Determination of Muscle Texture Characteristics

At room temperature, the samples were cut into dimensions of 2.0 cm × 1.5 cm × 1.0 cm and subjected to TPA using a fully automated high-precision texture analyzer (TBUTA-23, Tengba Instrument Technology Co., Ltd., Shanghai, China). All samples were measured in triplicate. The instrument conditions were operated following our previous study [21].

#### 2.2.2. Determination of Conventional Nutritional Components

Moisture content was determined by the direct drying method, where samples were dried at 103 °C for 12 h in a thermostatic electric blast drying oven (GZX-GF101-3-BS, Yuejin Medical Instrument Co., Ltd., Shanghai, China). Ash content was determined using the muffle furnace ignition method, where samples were incinerated at 550 °C for 4 h in a muffle furnace (B180, Nabertherm GmbH, Lilienthal, Germany). Crude fat content was determined according to the Chinese national standard method for fat content determination [22]. Crude protein content was determined following the Chinese national standard method for protein content determination [23].

### 2.3. Determination of Fatty Acid and Amino Acid Composition

The content of 24 fatty acids in the samples was determined using an Agilent 7890 A gas chromatograph and mass spectrometer, following the method outlined in GB 5009.168-2016 [24]. The content of 16 amino acids in the samples was determined using a Hitachi L-8900 fully automated amino acid analyzer, following the method outlined in GB 5009.124-2016 [25].

Lipid extraction: Approximately 2 g of fish muscle was weighed and placed in a 50 mL centrifuge tube. A total of 10 mL of a chloroform–methanol solution (*v*/*v* = 2:1) was added, followed by vortexing for 1 min and sonication for 30 min. Subsequently, 10 mL of distilled water was added, vortexed for 30 s, and centrifuged at 8000 rpm for 10 min. The lower chloroform layer was collected, dried over anhydrous sodium sulfate, and evaporated under a nitrogen gas stream.

Fatty acid methylation: To the dried lipid extract, 2 mL of 0.5 mol/L KOH solution was added, and the sample was saponified under a nitrogen atmosphere in a water bath at 80 °C for 1 h. Then, 1 mL of 13% BF_3_ was added, and the mixture was vortexed to dissolve the residue. Nitrogen gas was introduced to cool the bottle. Methylation was carried out in a water bath at 80 °C for 30 min. Thereafter, 1 mL of *n*-hexane was added, and the mixture was vortexed. The upper layer was collected after passing the solution through an anhydrous sodium sulfate column for subsequent analysis.

GC conditions: An HP-5MS gas chromatography column (30 m × 0.25 mm, 0.25 μm) was used with splitless injection mode. The heating program was initiated at 100 °C and held for 13 min. The temperature was then increased at a rate of 10 °C/min to 180 °C, followed by a ramp of 1 °C/min to 200 °C, where it was held for 20 min. Finally, the temperature was increased at 4 °C/min to 230 °C and held for 10.5 min, resulting in a total run time of 49.5 min. The injector temperature was set at 270 °C. High-purity helium (99.999%) served as the carrier gas at a flow rate of 0.8 mL/min.

### 2.4. Nutritional Evaluation

The nutritional evaluation was conducted using the amino acid scoring standards recommended by the Food and Agriculture Organization/World Health Organization (FAO/WHO) and the whole egg protein amino acid scoring model proposed by the Nutrition and Food Safety Institute of the Chinese Center for Disease Control and Prevention. The calculations included the Amino Acid Score (AAS), Chemical Score (CS), and Essential Amino Acid Index (EAAI). The formulas are as follows:

AAS=AA1/AA2 × 100

CS=AA1/AA3 × 100

$$EAAI=\sqrt[{n}]{\left(100A/AE)\times (100B/BE)\times (100C/CE)...\times (100H/HE\right)}$$

where:

AA1: Amino acid content in sample protein
AA2: Amino acid content in scoring protein
AA3: Amino acid content in whole egg protein
n: The number of essential amino acids compared.
A, B, …H: The content of essential amino acids in muscle protein, expressed in mg/g protein.
AE, BE, …HE: The corresponding amino acid content in whole egg protein, expressed in mg/g protein.

### 2.5. Electronic Nose Analysis

Based on the method of Huiping W et al. [26], the odor characteristics of the samples were analyzed using an electronic nose fingerprint detection system (PEN3, AirSense Analytics, Schwerin, Germany). The performance specifications of the 10 sensors are detailed in Table 1. Prior to sample analysis, several blank measurements (BLK and SAMBLK) were conducted to ensure the system was free of background interference. No interfering signals were detected in the blank runs. Method validation confirmed that the system exhibited satisfactory analytical performance, with recovery rates of characteristic odor compounds ranging from 90% to 110%, and relative standard deviations (RSDs) below 10%, indicating good accuracy and reproducibility.

### 2.6. HS-SPME-GC-MS Analysis

Before the first use, the 75 μm CAR/PDMS extraction fiber was placed in the GC injection port at 250 °C under nitrogen protection and aged for 2 h. Subsequently, the fiber was activated at 250 °C for 30 min before each use. Several blank runs (BLK and SAMBLK) were performed under identical conditions without sample injection to ensure no interference peaks were detected by GC-MS. All blank tests confirmed the absence of contaminant peaks. Additionally, method validation showed that the recovery rates of the target compounds ranged between 90% and 110%, with relative standard deviations (RSDs) below 10%, indicating good accuracy and precision. The specific operating conditions are detailed in the Supplementary Materials.

### 2.7. Molecular Docking

Molecular docking analysis was conducted on the screened key differential volatile flavor compounds. The 3D structural models of the flavor compounds were obtained from the PubChem website (https://pubchem.ncbi.nlm.nih.gov/), and the 3D structural models of ORs were obtained from the UniProt website (https://www.uniprot.org/). The flavor compounds and ORs were preprocessed using methods such as hydrogenation and removal of water molecules. Discovery Studio 2019 software was used for molecular docking, and binding pockets were identified automatically. Molecular binding was determined from both 2D and 3D perspectives, and binding energy and hydrophobic interactions were recorded. For the details of the selected ORs, please refer to the Supplementary Materials Text S1.

### 2.8. Statistical Analysis

Statistical analysis was conducted using SPSS 23.0 (IBM Corp, Armonk, NY, USA). One-way analysis of variance (ANOVA) followed by Duncan’s multiple comparison tests was used to compare the significant differences between different groups. Results were presented as mean ± standard deviation (SD), with significance determined at *p* < 0.05. GraphPad Prism 8.0.1 was used to generate plots, while WinMuster (version 1.6.2.18) software was utilized for analyzing and visualizing electronic nose data. Multivariate regression analysis was conducted on the BioSciCloud and BioDeep platforms.

## 3. Results and Discussion

### 3.1. Muscle Texture and Proximate Composition

**Figure 1:**
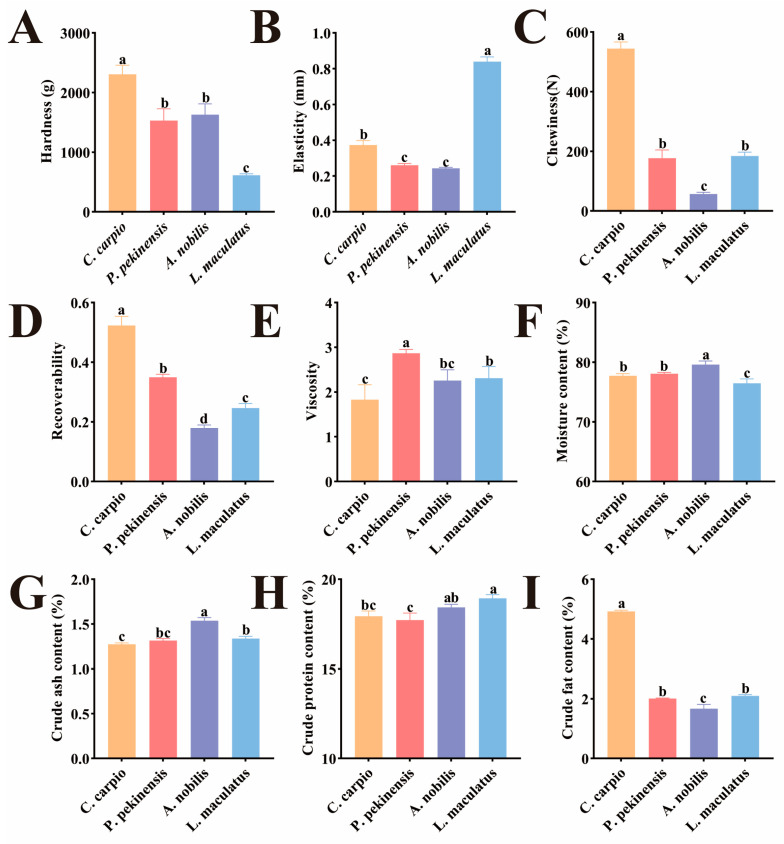
Muscle texture and proximate composition of different fish species. (**A**) Hardness. (**B**) Elasticity. (**C**) Chewiness. (**D**) Resilience. (**E**) Viscosity. (**F**) Moisture content. (**G**) Crude ash content. (**H**) Crude protein content. (**I**) Crude fat content. Different lowercase letters indicate significant differences between the tested muscle samples, *p* < 0.05.

The texture characteristics differed significantly among the four sample groups (Figure 1A–E). In terms of hardness, *C. carpio* showed significantly higher hardness than other fish species (*p* < 0.05), while *L. maculatus* had the lowest hardness (*p* < 0.05). Studies have shown that muscle fiber content is positively correlated with muscle hardness [27]. *C. carpio* has a larger turning angle during rapid starts and escapes, which requires greater muscle strain and inadvertently increases its muscle fiber content [28]. This may explain why *C. carpio* has higher hardness than other fish species. In terms of elasticity, *L. maculatus* was significantly higher than other fish species, while there was no significant difference between *P. pekinensis* and *A. nobilis*, but both were significantly lower than the other species (*p* < 0.05). The isometric growth characteristic of *L. maculatus* may result in its muscle tissue having higher elasticity [29]. The four fish species showed significant differences in chewiness and resilience (*p* < 0.05), with the order being *C. carpio* > *P. pekinensis* > *L. maculatus* > *A. nobilis*. Studies have shown that chewiness and resilience are significantly correlated with muscle hardness [30,31]. The trends of chewiness and resilience among the groups were almost consistent with the trend of hardness, indirectly confirming the accuracy of our experiment. Studies suggest that, compared to other fish species, the levels of iron and manganese in the muscle and gills of *P. pekinensis* are higher [32], which may contribute to the increased adhesiveness of its muscle.

Under normal circumstances, moisture, ash, crude protein, and crude fat are considered standard nutritional indicators for fish meat analysis. The moisture and ash content of *A. nobilis* were significantly higher than those of the other three fish species (*p* < 0.05) (Figure 1F,G). In terms of crude protein content, *L. maculatus* had the highest content (*p <* 0.05), while *P. pekinensis* had the lowest (Figure 1H). This is closely related to their dietary habits, as *L. maculatus*, a carnivorous fish, consumes the most protein, whereas *P. pekinensis*, an herbivorous fish, has limited access to animal protein [33,34]. Plant proteins generally have lower digestibility and biological value compared to animal proteins, with limitations in essential amino acids such as lysine and methionine [35]. Regarding crude fat, *C. carpio* had significantly higher levels than the other three fish species (*p* < 0.01), while *A. nobilis* had the lowest crude fat content (Figure 1I). During farming, economic motivations often lead to the addition of excessive starch in feed [36]. As an omnivorous fish, *C. carpio* has limited ability to absorb sugar, resulting in the accumulation of starch in its body, which increases its fat content.

### 3.2. Analysis of Muscle Fatty Acid Composition

Fish muscle contains a diverse composition of fatty acids, with a high content of unsaturated fatty acids. They help reduce the risk of atherosclerosis, heart disease, and cancer [37]. The composition and content of muscle fatty acids differ among *C. carpio*, *P. pekinensis*, *A. nobilis*, and *L. maculatus* (Table 2). *P. pekinensis* had the poorest fatty acid composition, with only 19 types. The other three species had the same number of fatty acid types but showed differences in their composition. Among them, significant differences in saturated fatty acid (SFA) enrichment were observed among the samples, with palmitic acid being the most abundant, ranked as *A. nobilis* > *C. carpio* > *P. pekinensis* > *L. maculatus* (*p* < 0.05). SFAs are considered to raise serum cholesterol levels and increase the risk of cardiovascular diseases [38]. Therefore, from the perspective of SFA enrichment, the consumption of *L. maculatus* is more beneficial to human health. Significant differences in monounsaturated fatty acid (MUFA) content were observed among the four groups, with oleic acid being the most abundant, ranked as *L. maculatus* > *C. carpio* > *P. pekinensis* > *A. nobilis* (*p* < 0.05). MUFA influences health, well-being, and disease risk by affecting intracellular signaling pathways, transcription factor activity, and gene expression [39]. This indicates that compared to the muscles of the other three fish species, *L. maculatus* is a superior source of MUFA. The polyunsaturated fatty acid (PUFA) content also showed differences among the four groups, with linoleic acid, eicosapentaenoic acid (EPA), and docosahexaenoic acid (DHA) being the primary contributors. The order was *P. pekinensis* > *A. nobilis* > *C. carpio* > *L. maculatus*. Previous studies have shown that PUFA has various positive effects on human health, including improving cardiovascular health, promoting fat metabolism, enhancing body composition, and supporting neural and cognitive development [40]. Therefore, *P. pekinensis* can be considered a high-quality source of PUFA for human consumption. Additionally, the FAO/WHO dietary standards suggest that an n-3/n-6 PUFA ratio of at least 0.1–0.2 in fatty acid composition can reduce the risk of cardiovascular diseases [41]. In this study, all fish species met this standard.

### 3.3. Analysis of Muscle Amino Acid Composition

Typically, the amino acids in fish muscle can be categorized into essential amino acids (EAAs), non-essential amino acids (NEAAs), and delicious amino acids (DAAs) [42]. EAAs and NEAAs play a critical role in maintaining human health, preventing diseases, and promoting growth and development, while DAAs, as the source of the “taste” in fish muscle, can significantly influence the flavor of fish meat [43]. A total of 17 amino acids were detected in the muscle samples of all four fish groups, including 8 EAAs and 9 NEAAs, among which 4 were DAAs. The amino acids with higher content were glutamic acid, lysine, aspartic acid, and leucine, in that order; however, the proportions of amino acids varied across different fish muscle samples (Table 3). FAO/WHO proposed that when the ratio of EAAs to TAAs is around 40% and EAAs/NEAAs is ≥60%, the protein quality is considered good, meeting the ideal model for balanced protein needs in humans [41]. In this study, all four muscle sample groups met this standard and can be considered high-quality protein sources. Meanwhile, AAS and CS were introduced to evaluate the nutritional quality of the muscle. Based on the AAS evaluation, the first limiting amino acid in all four muscle samples was valine. The second limiting amino acid was phenylalanine and tyrosine for *A. nobilis*, *C. carpio*, and *P. pekinensis*, while for *L. maculatus*, it was threonine. According to the CS evaluation, the first limiting amino acid in all four groups was phenylalanine and tyrosine, and the second limiting amino acid was methionine and cysteine. Therefore, in practical aquaculture production, appropriate supplementation with limiting amino acids can improve feed nutritional value and enhance fish production [44]. It is noteworthy that tryptophan was not detected. This is because its chemical structure is degraded under acidic hydrolysis conditions, making it undetectable by traditional methods. For future studies requiring accurate determination of tryptophan, we suggest adopting some improved methods, such as adding thiol antioxidants like mercaptoacetic acid or phenol in the hydrolysis system, or replacing hydrochloric acid with p-toluenesulfonic acid during the hydrolysis process to improve tryptophan recovery and ensure accurate quantification.

In conclusion, by integrating texture and nutritional data, a comprehensive “breed-to-market” optimization strategy can be established to enhance the added value and market competitiveness of freshwater fish products. *L. maculatus*, with its high elasticity, high protein content, and elevated MUFA levels, is particularly suitable for developing high-end raw products, such as sushi, or products requiring shape retention, such as cold-smoked fish. *C. carpio*, characterized by its high hardness and adhesive properties, is ideal for products like fish balls or shaped fried items. *P. pekinense*, with its high PUFA levels, including DHA and EPA, is well-suited for functional fish oil extraction or the development of minimally processed, health-focused food items. *A. nobilis*, with its unique parameters, is recommended for use as a seasoning ingredient in ready-to-cook products or for the production of smoked specialty items, such as smoked fish slices.

### 3.4. Electronic Nose Detection and Volatile Compound Analysis

**Figure 2:**
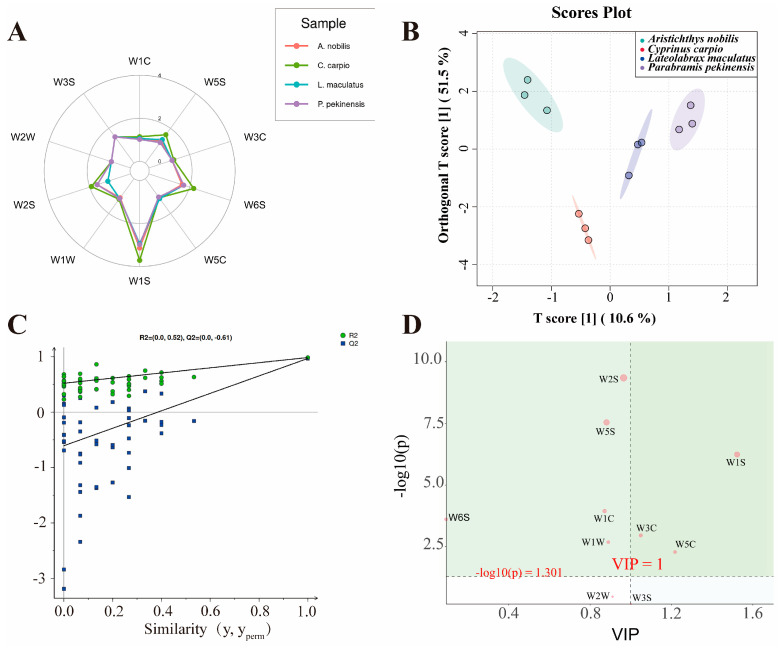
Detection using electronic nose technology and analysis of volatile flavor compounds. (**A**) Radar chart showing the distribution of key variables across four sample groups. (**B**) OPLS-DA score plot demonstrating the separation of sample groups based on response values. The ellipses represent the 95% confidence intervals. (**C**) Permutation test plot of the OPLS-DA model indicating the robustness of the model. R^2^ and Q^2^ values are shown for each permutation. (**D**) S-plot from the OPLS-DA analysis showing variables contributing to group separation. Red-labeled points indicate significant variables (VIP > 1 and *p* < 0.05 after FDR correction).

In all muscle tissue samples, the response values of the seven sensors W5C, W3C, W5S, W1C, W3S, W2W, and W1W were relatively close, indicating that the overall volatile compounds detected in the muscle samples were generally similar (Figure 2A). Additionally, the overall response values were highest for the W1S, W6S, and W2S sensors, which, to some extent, indicates that methyl compounds, hydrocarbons, alcohols, aldehydes, and ketones significantly contributed to the volatile odor of the muscle. Notably, the samples exhibited good differentiation on the W1S sensor, providing a useful reference for distinguishing the volatile flavor compounds of these four fish.

The orthogonal partial least squares-discriminant analysis (OPLS-DA) model is a multivariate projection method used to analyze correlations, differentiation, and effect changes in data [45]. Compared to PCA and PLS-DA, it provides better model fit, diagnostic capability, and interpretability, making it particularly suitable for identifying components and properties in food detection. The muscle samples of each group exhibited good inter-group dispersion and intra-group clustering (Figure 2B), consistent with the results of previous proximate composition and amino acid component analyses, indirectly validating the accuracy of earlier experiments. Additionally, the OPLS-DA permutation test plot (Figure 2C) was used to validate the model’s fitting accuracy and interpretability. R^2^X represents the model’s explanatory power for predictor variables, R^2^Y represents its explanatory power for response variables, and Q^2^ represents the model’s predictive ability. The values of these parameters range from 0 to 1, with 1 indicating the strongest capability [46,47]. The results showed that all blue Q^2^ points on the left are lower than the original blue Q^2^ point on the far right, and the OPLS-DA model achieved R^2^X = 0.94, R^2^Y = 0.985, and Q^2^ = 0.968. This indicates that the OPLS-DA model exhibits excellent fitting accuracy and interpretability. To further investigate the types of volatile flavor compounds with significant contributions in all samples, S-plot was employed to further screen the results of the OPLS-DA model. The S-plot model is primarily used in electronic nose food detection to identify and distinguish key chemical fingerprint markers, thereby effectively assessing food quality, safety, and flavor characteristics [48,49]. Points at the ends of the “S” represent potential chemical markers with high confidence. Variables with smaller contributions to classification cluster near the origin, while those with larger contributions are distributed at the ends of the “S” shape [50]. W1S, W2S, and W5S were the differential electronic nose sensors that contributed most to the volatile flavor of muscle samples (Figure 2D). These three electronic nose sensors can be used in future studies as one of the standards to distinguish and differentiate these four fish and to identify food fraud.

### 3.5. HS-SPME-GC-MS Analysis and Volatile Compound Profiling

**Figure 3:**
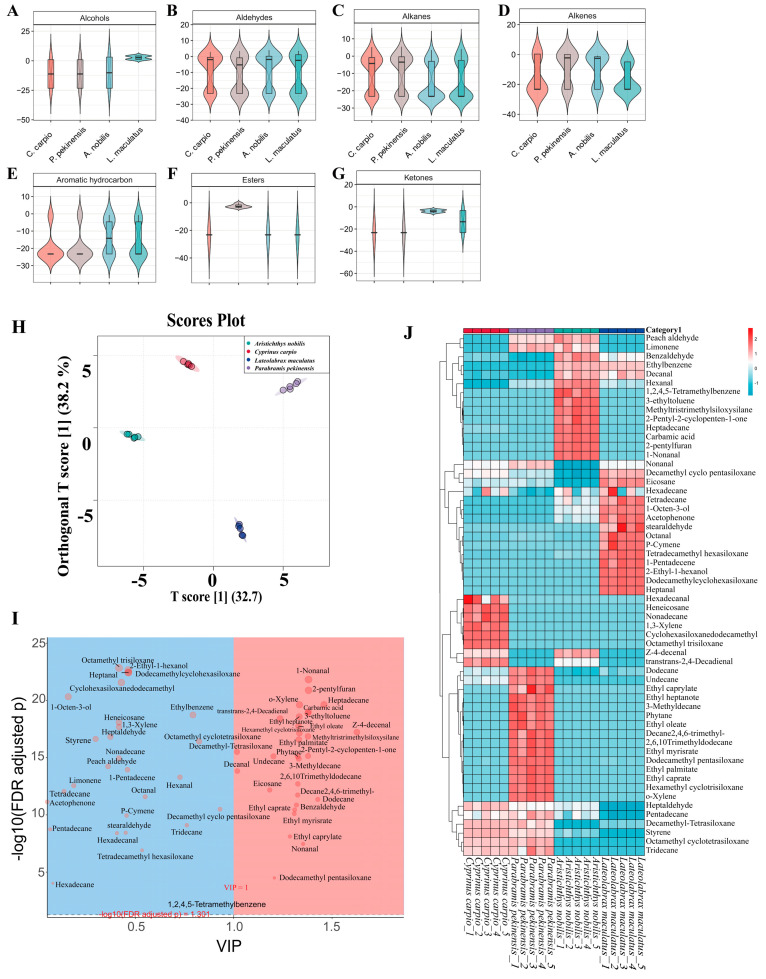
Visualization analysis of flavor compounds in samples. (**A**) Alcohols. (**B**) Aldehydes. (**C**) Alkanes. (**D**) Alkenes. (**E**) Aromatic hydrocarbons. (**F**) Ketones. (**G**) Phenols. (**H**) OPLS-DA score plot showing separation among species based on their volatile profiles. Each point represents an individual sample, and the ellipses indicate 95% confidence intervals. (**I**) Variable importance in projection (VIP) plot derived from the OPLS-DA model, highlighting differential metabolites (VIP > 1, FDR-adjusted *p* < 0.05). Red and blue areas denote significantly enriched compounds in different sample groups. (**J**) Heatmap of selected volatile compounds with hierarchical clustering. Rows represent metabolites, and columns represent biological replicates. The color scale indicates relative abundance (red: high; blue: low).

To further investigate the key volatile flavor compounds of *C. carpio*, *P. pekinensis*, *A. nobilis*, and *L. maculatus*, a total of 59 volatile flavor compounds were detected and screened using HS-SPME-GC-MS technology and the NIST spectral library. Various mathematical models were constructed to systematically analyze the experimental results. The alcohol content in *L. maculatus* was significantly higher than in the other groups (*p* < 0.05) (Figure 3A). Alcoholic volatile flavor compounds enhance the overall flavor of fish meat and contribute a mushroom-like aroma to some extent [51]. They are primarily derived from unsaturated fatty acids, consistent with the earlier GC-MS findings showing *L. maculatus* had the highest MUFA content. There were no significant differences in aldehyde volatile flavor compounds among the sample groups, except that *P. pekinensis* had slightly lower aldehyde content compared to the other samples (Figure 3B). Aldehyde compounds can contribute a pleasant fruity aroma at low concentrations [52], which might explain the unique flavor of *P. pekinensis*. Regarding alkane content, *A. nobilis* and *L. maculatus* had significantly lower alkane levels than the other samples (Figure 3C, *p* < 0.05). Although there were large differences in alkane content among the groups, the high threshold of alkane compounds meant they had little impact on the overall flavor of the fish meat [53]. For alkene content, *P. pekinensis* and *A. nobilis* had significantly higher levels than *C. carpio* and *L. maculatus* (Figure 3D, *p* < 0.05). Alkene compounds mainly originate from two sources: green forage feeding and accumulation in the animal’s fat reserves [54,55]. Alkenes have characteristic aromas of pine, camphor, lemon, and flowers, making a significant contribution to flavor [56]. The aromatic hydrocarbon and ketone content of *A. nobilis* was significantly higher than that of the other three groups (*p* < 0.05) (Figure 3E,G). Aromatic hydrocarbons have a low threshold and a strong meaty aroma [57]; ketones, with their low threshold, impart a unique milky and fatty aroma to the meat [58]. These volatile flavor compounds collectively contribute to the distinctive flavor of *A. nobilis*. Additionally, ester compounds also significantly contribute to the flavor of fish meat [53]. These compounds are typically formed through the esterification of carboxylic acids and alcohols derived from lipid metabolism. The ester content of *A. nobilis* was significantly higher than that of the other groups (*p* < 0.05) (Figure 3F), which might be attributed to its unique feeding habits.

To identify the specific volatile flavor compound markers that significantly contribute to the muscle samples of *C. carpio*, *P. pekinensis*, *A. nobilis*, and *L. maculatus*, an OPLS-DA model was constructed to further analyze the experimental data. The four sample groups showed significant inter-group dispersion and good intra-group clustering (R^2^X = 0.708, R^2^Y = 0.996, Q^2^ = 0.994), indicating excellent fitting accuracy, diagnostic capability, and interpretability of the OPLS-DA model (Figure 3H). Furthermore, 29 volatile flavor compounds with significant contributions were identified based on multiple criteria (VIP > 1, “S-plot” confidence level, and *p* < 0.01) (Figure 3I). These volatile flavor compounds include z-4-decenal, heptadecane, dodecane, 2-pentylfuran, 1-nonanal, carbamic acid, 2-pentyl-2-cyclopenten-1-one, methyltris(trimethylsiloxy)silane, 3-ethyltoluene, nonanal, o-xylene, hexamethyl cyclotrisiloxane, ethyl heptanoate, ethyl palmitate, 3-methyldecane, ethyl oleate, 2,6,10-trimethyl-dodecane, 2,4,6-trimethyldecane, phytane, benzaldehyde, ethyl caprate, ethyl myristate, ethyl caprylate, trans,trans-2,4-decadienal, dodecamethyl-pentasiloxane, undecane, eicosane, and decanal. These volatile flavor compounds can serve as key biomarkers shared by *C. carpio*, *P. pekinensis*, *A. nobilis*, and *L. maculatus*.

To further differentiate the key volatile flavor compounds among *C. carpio*, *P. pekinensis*, *A. nobilis*, and *L. maculatus* populations and investigate the differences in their volatile flavor compounds, a correlation clustering heatmap was used to illustrate sample differences and achieve classification of the target samples. The differences in volatile flavor compounds among the sample groups were significant (Figure 3J). 1,3-xylene, cyclohexasiloxane, dodecamethyl, heneicosane, hexadecanal, nonadecane, and octamethyl-trisiloxane can serve as volatile differential markers for *C. carpio*, with 1,3-xylene imparting a rich geranium aroma to the fish [59]. 2,6,10-trimethyl-dodecane, 3-methyldecane, 2,4,6-trimethyldecane, dodecamethyl-pentasiloxane, ethyl caprate, ethyl caprylate, ethyl heptanoate, ethyl myristate, ethyl oleate, ethyl palmitate, hexamethyl cyclotrisiloxane, o-xylene, and phytane can serve as volatile differential markers for *P. pekinensis*. Among these, ethyl esters possess rich floral and fruity aromas [26], which may be a key source of *P. pekinensis’* pleasant aroma. 1,2,4,5-tetramethylbenzene, 1-nonanal, 2-pentyl-2-cyclopenten-1-one, 2-pentylfuran, 3-ethyltoluene, carbamic acid, heptadecane, hexanal, and methyltris(trimethylsiloxy)silane can serve as volatile differential markers for *A. nobilis*. When mixed, 1-nonanal and hexanal not only produce a pleasant fruity aroma but also introduce some grassy notes [60]; 2-pentylfuran, as a non-carboxylic compound, generates a pronounced meaty aroma [55]. 1-pentadecene, 2-ethyl-1-hexanol, cyclohexasiloxane, dodecamethyl, heptanal, octanal, p-cymene, stearaldehyde, and tetradecamethyl hexasiloxane can serve as volatile differential markers for *L. maculatus*. Among these, heptanal and octanal contribute to a rich blend of aromas for *L. maculatus*, such as nutty, roasted, and fatty notes [61,62].

In summary, the four fish species exhibit distinct characteristics. The flavor of *C. carpio* is dominated by alkanes, resulting in a mild and gentle taste, making it particularly suitable for consumers who prefer light, delicate, and smooth mouthfeels. *P. pekinensis*, on the other hand, is characterized by a distinct presence of aldehyde compounds, which contribute to its fresh and fruity aroma, catering to consumers who favor pronounced fruity notes. *A. nobilis* exhibits a rich flavor profile primarily composed of aromatic hydrocarbons and ketones, delivering intense meaty and dairy-like aromas, making it ideal for those who enjoy bold and complex flavors. *L. maculatus* is rich in volatile alcohol compounds, which impart a subtle and layered mushroom-like aroma, appealing to consumers who prefer tender, nuanced, and sophisticated textures and flavors.

### 3.6. Molecular Docking Between Key Differential Volatile Flavor Compounds and ORs

OR1A1, OR1D2, OR2J3, and OR2W1 were selected as the target proteins for molecular docking. OR1A1, a member of the OR1 family, plays a key role in detecting a wide range of odorants and is predominantly expressed in olfactory sensory neurons, making it highly relevant for studying the interaction between flavor compounds and fish muscle [63]. OR1D2 is involved in perceiving various volatile compounds, particularly aldehydes and other carbonyl compounds, which contribute significantly to the flavor profile of fish [64]. OR2J3, part of the OR2 family, detects amines and other nitrogenous compounds found in fish muscle, influencing its odor and flavor [65]. OR2W1, another OR2 family member, responds to terpenoids and other scent molecules, potentially contributing to the natural aroma of fish muscle and enhancing our understanding of its sensory perception [66].

**Figure 4:**
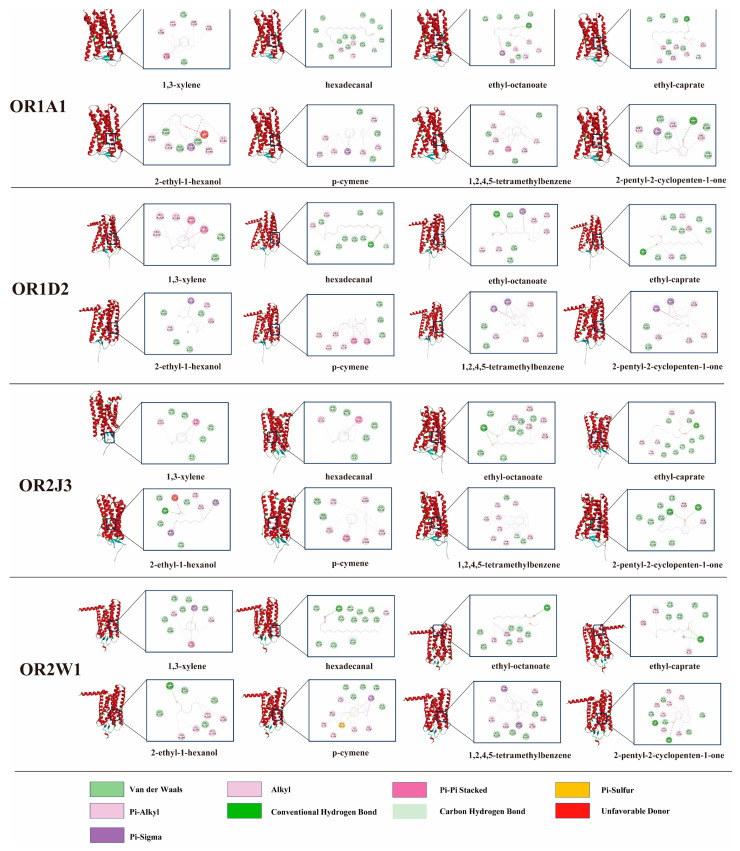
2D and 3D conformations of ORs and flavor compounds after docking. Specifically, the red protein conformations represent the 3D structure, while the 2D connection models are shown in the right-side box.

To elucidate the mechanistic basis of these molecular interactions, complexes exhibiting the lowest binding energies were selected and visualized (Figure 4). Binding energies below 0 kcal/mol were considered indicative of spontaneous molecular interactions [67]. The calculated binding energies between four ORs and flavor compounds ranged from −7.5 kcal/mol to −4.7 kcal/mol (Table S1). Mean binding energies for key volatiles—hexadecanal, 1,3-xylene, ethyl octanoate, ethyl caprate, p-cymene, 2-ethyl-1-hexanol, 1,2,4,5-tetramethylbenzene, and 2-pentyl-2-cyclopenten-1-one—were determined as −6.175, −5.625, −5.4, −5.65, −6.475, −5.0, −6.2, and −6.025 kcal/mol, respectively. Higher binding energies correlated with weaker ligand–receptor affinities. Notably, p-cymene demonstrated the lowest mean binding energy (−6.475 kcal/mol). Its cyclic aromatic structure, featuring a conjugated double bond and methyl substituent, facilitates π–π stacking interactions with aromatic residues within the OR binding pocket. Conversely, 2-ethyl-1-hexanol exhibited the weakest affinity (−5.0 kcal/mol), likely attributable to steric hindrance caused by its extended alkyl chain. The increased molecular volume potentially restricts optimal contact with the receptor’s binding site, thereby destabilizing the interaction.

Hydrogen bonding with ORs was observed for all flavor compounds except 1,3-xylene and p-cymene. This absence was attributed to their classification as aromatic hydrocarbons lacking heteroatoms (e.g., oxygen or nitrogen) and polar functional groups necessary for hydrogen bond donation or acceptance [68]. Recurrent involvement of specific amino acid residues across interactions suggested their critical role as binding anchors. Notably, ILE105 in OR1A1 and TYR259 in OR1D2 demonstrated hydrophobic interactions with all eight flavor compounds. These recurring residues likely function as conserved recognition sites governing ligand–receptor binding specificity.

Hydrogen bonding was shown to enhance interactions between flavor compounds and ORs [69]. Ethyl caprate formed two hydrogen bonds with OR1A1 and three with OR1D2 (Table S2), potentially explaining its lower binding energy to OR1A1 compared to OR1D2. However, hydrogen bond quantity did not strictly correlate with binding affinity. Notably, p-cymene exhibited the lowest binding energy (−6.475 kcal/mol) despite lacking hydrogen bonds with OR1D2. This phenomenon was attributed to π–π stacking interactions between p-cymene and aromatic residues (PHE and TYR) within the binding pocket (Figure 4). π–π stacking, a non-covalent interaction characteristic of aromatic systems, arises from parallel alignment of π-electron orbitals, enhancing molecular contact area and stabilizing binding [70]. The hydrophobic nature of this interaction further complemented the receptor’s non-polar microenvironment, promoting stable complex formation. These findings align with prior studies emphasizing the significance of hydrophobic forces alongside hydrogen bonding [71,72]. Future investigations should employ integrated experimental and computational approaches (e.g., molecular dynamics simulations, quantum chemical calculations) to systematically evaluate additional non-covalent interactions, including π–sigma and cation–π interactions, in odorant–OR recognition mechanisms.

## 4. Conclusions

This study investigated muscle quality and flavor differences among *C. carpio*, *P. pekinensis*, *L. maculatus*, and *A. nobilis* by analyzing textural properties, nutritional profiles, and molecular sensory techniques. Significant differences were observed in texture, with *C. carpio* exhibiting the highest hardness and *L. maculatus* demonstrating superior elasticity, reflecting their ecological adaptations. Nutritional profiling identified *L. maculatus* as a high-quality protein source, while *C. carpio* had higher crude fat levels. Fatty acid analysis highlighted the richness of *L. maculatus* in MUFA and *P. pekinensis* in PUFA, corresponding to their dietary niches. Additionally, 59 key flavor biomarkers were identified, including aldehydes, alcohols, and aromatic hydrocarbons, which defined species-specific flavor profiles. Molecular docking revealed critical interactions between flavor compounds and olfactory receptors, emphasizing the role of π–π stacking and hydrophobic forces in flavor perception. These findings provide insights for optimizing aquaculture management, improving feed formulations, and aligning production practices with consumer preferences, supporting the sustainable development of freshwater species.

## Figures and Tables

**Table 1:** The performance description of the 10 sensors in the PEN3 electronic nose.

Sensor	Substances for Sensing
W1C	Aromatic benzene
W5S	Nitrogen oxides
W3C	Aromatic ammonia
W6S	Hydrogen
W5C	Alkane aromatic compounds
W1S	Short-chain alkanes
W1W	Sulfides and terpenes
W2S	Alcohols, aldehydes, and ketones
W2W	Organic sulfides and aromatic components
W3S	Long-chain alkanes

**Table 2:** Differences in fatty acid composition.

Fatty Acids (g/100 g Total Fatty Acid)	*C. carpio*	*P. pekinensis*	*A. nobilis*	*L. maculatus*
Lauric acid (C12:0)	0.03 ± 0.01 b	-	0.15 ± 0.03 a	-
Tridecanoic acid (C13:0)	-	-	-	-
Myristic acid (C14:0)	1.47 ± 0.12 b	1.30 ± 0.12 c	5.69 ± 0.02 a	0.68 ± 0.03 d
Pentadecanoic acid (C15:0)	0.50 ± 0.01 b	0.08 ± 0.02 c	1.01 ± 0.04 a	0.11 ± 0.01 c
Palmitic acid (C16:0)	20.27 ± 2.06 a	19.05 ± 1.13 a	18.99 ± 0.83 a	14.16 ± 0.74 b
Heptadecanoic acid (C17:0)	0.54 ± 0.09 b	0.45 ± 0.07 b	1.12 ± 0.07 a	-
Stearic acid (C18:0)	6.30 ± 0.56 a	5.80 ± 0.10 a	4.68 ± 0.60 b	3.58 ± 0.14 c
Arachidic acid (C20:0)	0.20 ± 0.05 b	-	0.47 ± 0.01 a	0.23 ± 0.02 b
Heneicosanoic acid (C21:0)	0.21 ± 0.04 b	-	-	0.56 ± 0.03 a
Behenic acid (C22:0)	0.07 ± 0.02 b	0.12 ± 0.02 b	0.24 ± 0.07 a	0.30 ± 0.04 a
Myristoleic acid (C14:1)	-	-	-	-
Palmitoleic acid (C16:1)	7.72 ± 0.23 b	7.10 ± 0.27 c	9.20 ± 0.41 a	3.50 ± 0.11 d
Heptadecenoic acid (C17:1)	0.30 ± 0.07 a	0.12 ± 0.01 b	-	0.12 ± 0.01 b
Oleic acid (C18:1)	23.87 ± 0.91 b	18.64 ± 0.35 c	13.17 ± 1.16 d	37.53 ± 1.08 a
Eicosenoic acid (C20:1)	1.38 ± 0.27 b	0.54 ± 0.01 c	0.53 ± 0.10 c	2.13 ± 0.04 a
Erucic acid (C22:1n9)	-	-	0.03 ± 0.01 b	0.73 ± 0.06 a
Nervonic acid (C24:1)	-	-	0.14 ± 0.04 a	0.13 ± 0.03 a
Linoleic acid (C18:2n6c)	15.96 ± 1.13 a	11.45 ± 0.21 b	6.66 ± 1.03 c	10.90 ± 0.21 b
Gamma-linolenic acid (C18:3n6)	0.19 ± 0.01 c	0.06 ± 0.02 d	0.50 ± 0.05 a	0.36 ± 0.01 b
Alpha-linolenic acid (C18:3n3)	3.31 ± 0.29 c	9.92 ± 0.35 a	7.59 ± 0.73 b	1.73 ± 0.03 d
Eicosadienoic acid (C20:2)	0.94 ± 0.11 a	0.52 ± 0.03 c	0.49 ± 0.06 c	0.66 ± 0.04b
Eicosatrienoic acid (C20:3n3)	0.22 ± 0.01 c	1.02 ± 0.06 a	0.66 ± 0.07 b	0.14 ± 0.01 d
Di-γ-linolenic acid (C20:3n6)	0.85 ± 0.05 b	1.47 ± 0.07 a	0.78 ± 0.21 b	1.57 ± 0.05 a
Arachidonic acid (C20:4n6)	4.80 ± 0.41 b	5.98 ± 0.21 a	5.68 ± 0.82 ab	2.82 ± 0.13 c
EPA (C20:5n3)	5.61 ± 0.14 b	5.24 ± 0.02 b	9.49 ± 1.41 a	0.23 ± 0.01 c
DHA (C22:6n3)	5.18 ± 0.43 c	11.19 ± 0.30 b	13.20 ± 1.28 a	2.03 ± 0.11 d
∑SFA	29.57 ± 1.84 b	26.80 ± 1.04 c	32.35 ± 1.89 a	19.62 ± 0.74 d
∑MUFA	33.27 ± 1.01 b	26.40 ± 0.40 c	23.06 ± 1.34 d	44.16 ± 0.48 a
∑PUFA	37.06 ± 3.14 a	46.85 ± 0.36 a	45.06 ± 10.2 a	20.44 ± 0.18 b
∑(n − 3) PUFA	14.32 ± 0.36 c	27.37 ± 0.62 b	30.94 ± 0.33 a	4.13 ± 0.13 d
∑(n − 6) PUFA	21.79 ± 1.34 a	18.95 ± 0.21 b	13.62 ± 0.41 d	15.65 ± 0.41 c
∑(n − 3) PUFA/∑(n − 6) PUFA	0.66 ± 0.13 c	1.44 ± 0.05 b	2.27 ± 0.01 a	0.26 ± 0.04 d

Different lowercase letters indicate significant differences between the tested muscle samples, *p* < 0.05.

**Table 3:** Amino acid composition of the four sample groups.

Amino Acids	Content (mg/g)				Taste Attribute
	*C. carpio*	*P. pekinensis*	*A. nobilis*	*L. maculatus*	
Threonine *	0.82 ± 0.01 c	1.13 ± 0.01 a	0.75 ± 0.01 b	0.94 ± 0.01 d	Sweet
Valine *	0.97 ± 0.01 c	1.34 ± 0.01 a	0.91 ± 0.06 d	1.10 ± 0.01 b	Bitter
Methionine *	0.58 ± 0.01 c	0.81 ± 0.01 a	0.54 ± 0.02 d	0.68 ± 0.01 b	Bitter
Isoleucine *	0.87 ± 0.04 c	1.21 ± 0.04 a	0.82 ± 0.03 c	1.04 ± 0.04 b	Bitter
Leucine *	1.68 ± 0.06 c	2.40 ± 0.05 a	1.57 ± 0.01 d	1.95 ± 0.01 b	Bitter
Phenylalanine *	0.68 ± 0.01 c	0.91 ± 0.02 a	0.58 ± 0.01 d	0.76 ± 0.04 b	Bitter
Lysine *	1.86 ± 0.01 c	2.61 ± 0.01 a	1.75 ± 0.01 d	2.12 ± 0.02 b	Tasteless
Aspartic acid +	1.81 ± 0.01 c	2.56 ± 0.01 a	1.66 ± 0.02 d	2.11 ± 0.02 b	Umami
Glutamic acid +	2.65 ± 0.01 c	3.78 ± 0.03 a	2.53 ± 0.01 d	3.08 ± 0.01 b	Umami
Glycine +	1.13 ± 0.04 c	1.44 ± 0.01 a	0.93 ± 0.01 d	1.15 ± 0.02 b	Sweet
Alanine +	1.21 ± 0.01 c	1.64 ± 0.01 a	1.05 ± 0.04 d	1.33 ± 0.01 b	Sweet
Histidine ^	0.51 ± 0.00 a	0.52 ± 0.01 a	0.37 ± 0.02 b	0.34 ± 0.05 b	Bitter
Arginine ^	1.17 ± 0.04 c	1.69 ± 0.05 a	1.09 ± 0.02 d	1.31 ± 0.03 b	Sweet
Serine	0.74 ± 0.02 c	1.04 ± 0.02 a	0.69 ± 0.01 d	0.86 ± 0.01 b	Sweet
Cysteine	0.12 ± 0.01 d	0.18 ± 0.01 b	0.20 ± 0.01 a	0.14 ± 0.01 c	Tasteless
Tyrosine	0.51 ± 0.03 d	0.75 ± 0.04 a	0.51 ± 0.02 c	0.63 ± 0.02 b	Bitter
Proline	0.51 ± 0.01 b	0.63 ± 0.01 a	0.53 ± 0.03 b	0.53 ± 0.01 b	Tasteless
∑EAA	7.45 ± 0.14 c	10.41 ± 0.21 a	6.92 ± 0.17 b	8.59 ± 0.27 d	–
∑DAA	6.8 ± 0.33 c	9.42 ± 0.12 a	6.17 ± 0.14 d	7.67 ± 0.51 b	–
∑NEAA	10.37 ± 0.22 c	14.23 ± 0.09 a	9.56 ± 0.31 d	11.48 ± 0.29 b	–
∑TAA	17.82 ± 0.31 c	24.64 ± 0.33 a	16.48 ± 0.34 d	20.07 ± 0.25 b	–
∑EAA/∑TAA	0.42 ± 0.01 ab	0.42 ± 0.01 b	0.42 ± 0.01 ab	0.43 ± 0.01 a	–
∑DAA/∑TAA	0.38 ± 0.01 a	0.38 ± 0.02 a	0.37 ± 0.01 a	0.38 ± 0.01 a	–
∑EAA/∑NEAA	0.72 ± 0.02 b	0.73 ± 0.01 ab	0.72 ± 0.02 b	0.75 ± 0.01 a	–
Isoleucine	AAS	1.22	1.23	1.24	1.3
	CS	1.00	1	1.01	1.06
Leucine	AAS	1.34	1.39	1.36	1.39
	CS	1.16	1.2	1.18	1.2
Lysine	AAS	1.9	1.87	1.93	1.92
	CS	1.58	1.56	1.61	1.6
Threonine	AAS	1.15	1.14	1.13	1.12
	CS	1.02	1.02	1.01	1.04
Valine	AAS	1.08	1.09	0.91	1.1
	CS	1.01	1	0.84	1.01
Methionine + Cysteine	AAS	1.12	1.14	1.28	1.16
	CS	0.84	0.85	0.96	0.87
Phenylalanine + Tyrosine	AAS	1.11	1.12	1.1	1.14
	CS	0.78	0.78	0.77	0.77
Essential Amino Acid Index (EAAI)	84.80	80.29	88.80	88.75	

* Essential amino acids; + Delicious amino acids; ^ Semi-essential amino acids. Different lowercase letters indicate significant differences between the tested muscle samples, *p* < 0.05.

## Data Availability

The original contributions presented in this study are included in the article/Supplementary Materials. Further inquiries can be directed to the corresponding author.

## Supplementary Materials

The following Supporting Information can be downloaded at: https://www.mdpi.com/article/10.3390/foods14132258/s1, Text S1. HS-SPME-GC-MS Analysis; Table S1. Binding energy of key biomarkers with ORs; Table S2. Summary of the interaction forces between key biomarkers and ORs.
