# Supplementary material for: Texture, Nutrition, and Flavor of Different Freshwater Fish Muscles: Comparative Study and Molecular Docking
Source: Foods. 2025 Jun 26;14(13):2258. doi: 10.3390/foods14132258 (PMC12249016; doi:10.3390/foods14132258)
Supplement: Supplementary file 1 [file foods-14-02258-s001.zip › foods-3679702-supplementary.pdf]

Supplementary Materials

Text S1. HS-SPME-GC-MS Analysis

Aging and heating program: The initial temperature is 40 ℃, maintained for 1.0 minute, then heated at a rate of 5 ℃/min to 300 ℃, maintained for 37.0 minutes, and shared for 90.0 minutes; Injection port temperature 250 ℃; The carrier gas is 99.999% high-purity helium gas; Flow rate of 0.8mL/min.

Weigh the sample (2.0g ± 0.05g) (accurate to 0.01g) into a 15.0mL headspace vial, add 5.0mL of pure water, 7% sodium chloride, and 7.0 μ L of internal standard (2-methyl-3-heptanone, mass concentration 0.272 μ g/μ L), seal and vortex at 800r/min for 2.0 minutes. After vortexing, the sample was placed in a water bath and heated at a constant temperature of 100 ℃ for 30.0 minutes. It was then taken out and equilibrated in an 80 ℃ water bath for 10.0 minutes before extraction. Insert aged 75 μ m CAR/PDMS extraction fibers into the headspace section of a headspace bottle for suspended enrichment. After a certain extraction time, remove the extraction fibers and test them on the machine. The sample was repeated 3 times, and 3 groups of blank parallel control groups without added samples were conducted simultaneously.

GC conditions: DB-5MS gas chromatography column (30m × 0.25mm, 0.25 μ m); The entire mode adopts non split injection; Heating program: Initial temperature of 40 ℃, hold for 3.0min, then heat up at a rate of 4 ℃/min to 170 ℃, continue to heat up at a rate of 8 ℃/min to 230 ℃, hold for 5.0min, and then heat up at a rate of 20 ℃/min to 280 ℃, hold for 5.0min, totaling 60.5min; Injection port temperature 250 ℃; The carrier gas is 99.999% high-purity helium gas; Flow rate of 0.8mL/min.

MS condition: ionization mode EI; Ion source temperature 230 ℃; Quadrupole

rod temperature 150 ℃; Electron energy of 70eV; The scanning method is full scan; The quality scanning range is m/z 30-500 amu.

The composition of the tested component was determined by searching the NIST 08 spectral library; This study only analyzed the search results of compounds with a similarity index (SI) greater than 80.

Table S1. Binding energy of key biomarkers with ORs

Ligands	Binding energy (kcal/mol)			
	**OR1A1**	**OR1D2**	**OR2J3**	**OR2W1**
1,3-xylene	-5.2	-6.5	-5.4	-5.4
Hexadecanal	-6.5	-6.2	-6.1	-5.9
Ethyl-octanoate	-5.4	-5.9	-5.3	-5.0
ethyl-caprate	-5.8	-6.0	-5.7	-5.1
p-cymene	-6.0	-7.5	-5.9	-6.5
2-ethyl-1-hexanol	-4.7	-5.2	-4.8	-5.3
1,2,4,5-tetramethylbenzene	-5.9	-7.2	-5.9	-5.8
2-pentyl-2-cyclopenten-1-one	-5.8	-6.4	-5.8	-6.1

Table S2. Summary of the interaction forces between key biomarkers and ORs.

Receptor	Ligand	Hydrophobic interactions	Hydrogen bonds
OR1A1	1,3-xylene	ILE105, MET104, HIS159, MET199, PHE206, TYR258, ILE181	
	hexadecanal	VAL254, GLY108, TYR250, THR277, TYR258, PHE206, ILE105, ILE181, MET199, GLY202, HIS159, ILE205, ASN155, ASN109, MET104, TYR276, PHE73	ASN109
	ethyl-octanoate	HIS159, ILE105, ASN109, SER112, TYR250, GLY108, PHE73, TYR258, ILE181, VAL254, TYR276, PHE206, MET199	TYR250
	ethyl-caprate	GLY202, MET199, ILE105, ASN109, ILE181, MET104, GLY108, TYR250, ILE205, HIS159, PHE206, TYR276, VAL254	ASN109, TYR250
	2-ethyl-1-hexanol	PHE206, MET104, GLY108, ASN109, ILE105, VLA254, TYR258, MET199, ILE181	
	p-cymene	TYR258, MET104, ILE181, ILE105, ILE205, HIS159, PHE206, ASN155, ASN109, GLY202	
	1,2,4,5-tetramethylbenzene	GLY202, ASN109, MET104, ILE181, ILE205, PHE206, MET199, HIS159, TYR258, ILE105	
	2-pentyl-2-cyclopenten-1-one	ILE181, ILE105, MET104, ASN109, GLY108, TYR250, TYR258, PHE206, VAL254	ASN109
OR1D2	1,3-xylene	LEU208, LEU255, LEU199, PHE207, TYR259, TYR155, GLY203	
	hexadecanal	CYS256, LEU208, TYR252, LEU260, LEU255, PHE207, TYR259, GLY203, CYS204, ILE200, TYR182, LEU199	TYR182, LEU199
	ethyl-octanoate	ILE200, CYS204, TYR155, GLY203, TYR182, LEU199, TYR259, PHE207, TYR252, LEU255	TYR182
	ethyl-caprate	CYS204, PHE207, LEU208, LEU199, TYR259, TYR155, LEU255, CYS256, TYR252, ILE200, GLY203, TYR182	TYR182, ILE200, GLY203
	2-ethyl-1-hexanol	GLY203, CYS256, LEU260, LEU255, PHE207, TYR259, TYR252, LEU208	
	p-cymene	TYR252, LEU255, CYS256, LEU199, PHE207, TYR259, TYR182,	

		GLY203, TYR155	
	1,2,4,5-tetramethylbenzene	GLY203, LEU199, LEU208, TYR259, PHE207, LEU255, CYS256, TYR252	
	2-pentyl-2-cyclopenten-1-one	GLY203, LEU199, LEU208, TYR259, PHE207, LEU255, CYS256, TYR252	
OR2J3	1,3-xylene	LEU171, PHE15, ARG175, TYR97, SER11, GLY13, ALA9	
	hexadecanal	LEU171, PHE15, ARG175, TYR97, SER11, GLY13, ALA9	
	ethyl-octanoate	THR112, ASN158, LEU108, PHE209, TYR262, GLY111, VAL107, TYR76, CYS115, VAL210, PHE254, TYR281, ALA258	
	ethyl-caprate	PHE254, ALA258, TYR281, VAL210, CYS115, PHE209, THR112, ASN158, THR205, HIS162, GLY111, TYR262, LEU108, LEU202, SER206	SER206
	2-ethyl-1-hexanol	ASN158, THR112, SER206, TYR262, VAL210, LEU108, GLY111, TYR281, ALA158, PHE254	THR112
	p-cymene	PHE209, SER206, GLY111, TYR76, ALA258, VAL210, TYR262, TYR281, GLY111, PHE254	
	1,2,4,5-tetramethylbenzene	CYS115, VAL210, TYR76, ALA258, SER206, VAL107, TYR262, GLY111, LEU108, TYR281	TYR262
	2-pentyl-2-cyclopenten-1-one	LEU202, THR112, SER206, LEU208, VAL107, TYR76, PHE254, TYR262, TYR281, ALA258, GLY111	TYR262, TYR281
OR2W1	1,3-xylene	LEU159, GLY203. VAL199, VAL207, ASN155, ILE206, MET105, SER109, ILE255, TYR259	
	hexadecanal	PRO262, GLY263, GLN261, MET258, ASN264, TYR259, VAL199, LEU181, PRO182, VAL185, MET197, PHE200, GLU196	GLY263
	ethyl-octanoate	ASN137, MET136, PRO58, PRO138, CYS141, THR57, HIS56, PHE133, ARG64	ARG64
	ethyl-caprate	PRO58, PRO138, CYS141, THR57, PHE61, LEU55, TYR60, ARG64, HIS56	ARG64
	2-ethyl-1-hexanol	SER109, MET105, GLY108, GLY203, TYR259, VAL207, VAL199, ILE255, TYR104, LEU159	SER109

p-cymene	PHE251, GLY108, ASN155, SER109, TYR259, VAL207, GLY203, MET155, PHE73, CYS112, TYR104, ILE255, ILE206	
1,2,4,5-tetramethylbenzene	TYR259, CYS112, ILE206, GLY203, ASN155, LEU259, ILE255, LEU202, MET105, GLY108, SER109, VAL207, LEU159	
2-pentyl-2-cyclopenten-1-one	PHE73, TYR104, GLY203, TYR259, PHE251, LEU159, TYR278, SER109, ILE206, VAL207, ASN155, ILE255, MET105, GLY108	SER109, ASN155
